# Molecular Epidemiological Surveillance of Carbapenem-Resistant Gram-Negative Bacteria in Southern Lebanon

**DOI:** 10.3390/antibiotics14111124

**Published:** 2025-11-07

**Authors:** Anwar Al Souheil, Hadi Hussein, Ziad Jabbour, Sara Barada, Jose-Rita Gerges, Ghada Derbaj, Abdallah Kurdi, Hassan Jamil Kazma, Nour Nahouli, Ali Hasan Najem, Abdallah Medlej, Wael Zorkot, Rana El Hajj, Mahmoud I. Khalil, Ghassan M. Matar, Antoine Abou Fayad

**Affiliations:** 1 Department of Biological Sciences, Faculty of Science, Beirut Arab University, Beirut 1107 2809, Lebanon; anwaralsouheil@gmail.com (A.A.S.); m.khalil@bau.edu.lb (M.I.K.); 2 Department of Experimental Pathology, Immunology and Microbiology, Faculty of Medicine, American University of Beirut, Beirut 1107 2020, Lebanon; hh161@aub.edu.lb (H.H.); zj20@aub.edu.lb (Z.J.); sb133@aub.edu.lb (S.B.); jg39@aub.edu.lb (J.-R.G.); gd28@aub.edu.lb (G.D.); gmatar@aub.edu.lb (G.M.M.); 3 Center for Infectious Diseases Research, American University of Beirut, Beirut 1107 2020, Lebanon; 4 World Health Organization (WHO) Collaborating Center for Reference and Research on Bacterial Pathogens, Beirut 1107 2020, Lebanon; 5 Department of Biochemistry and Molecular Genetics, Faculty of Medicine, American University of Beirut, Beirut 1107 2020, Lebanon; ak161@aub.edu.lb; 6 Laboratory Department, Hammoud Hospital University Medical Center, Saida 11-2644, Lebanon; kazma.h@hotmail.com; 7 Laboratory Department, Al-Raee Hospital, Saida 1600, Lebanon; nournahouli@hotmail.com; 8 Laboratory Department, Lebanese Italian Hospital, Tyre 1645, Lebanon; dr.anajem@hotmail.com; 9 Molecular Biology Unit, Laboratory Department, Sheikh Ragheb Harb University Hospital, Nabatieh 11-2064, Lebanon; abdullahmedlij@gmail.com; 10 Faculty of Public Health, Lebanese University, Beirut 14-6573, Lebanon; zorkotwael.idmd@gmail.com; 11 Molecular Biology Unit, Department of Zoology, Faculty of Science, Alexandria University, Alexandria 21511, Egypt

**Keywords:** carbapenem-resistant Gram-negative bacteria, next-generation sequencing, antimicrobial resistance genes, plasmids

## Abstract

**Introduction:** Carbapenem-resistant Gram-negative bacteria (CR-GNB) are rapidly spreading pathogens that increase morbidity and mortality in hospital settings and significantly restrict available treatment options worldwide. The lack of molecular epidemiological data and the limited use of next-generation sequencing (NGS) in South Lebanon have hindered comprehensive surveillance efforts. This study represents the first molecular characterization of CR-GNB in this region. **Methods:** A total of 477 clinical Gram-negative bacterial isolates were collected from intensive care unit (ICU) patients admitted to various hospitals in South Lebanon in 2023. Of these, 131 CR-GNB were subjected to whole-genome sequencing using the Illumina MiSeq platform. K-mer-based species identification, multilocus sequence typing (MLST), antimicrobial resistance (AMR) gene profiling, and plasmid analysis were performed using multiple bioinformatic tools. Phylogenetic analysis was conducted using SaffronTree. **Results:** K-mer-based identification revealed that the predominant species among CR-GNB isolates were *Pseudomonas* spp. and *Escherichia coli* (26.7% each), followed by *Klebsiella pneumoniae* (19.8%), *Acinetobacter baumannii* (17.6%), *Proteus mirabilis* (4.6%), *Enterobacter cloacae* (2.3%), *Achromobacter* spp. (1.5%), and *Citrobacter freundii* (0.8%). Based on antimicrobial susceptibility testing, isolates were classified as follows: 0.8% as pan drug-resistant (PDR), 40.5% as extensively drug-resistant (XDR), and 52.7% as multidrug-resistant (MDR) and 6.1% as antimicrobial-resistant (AMR). All isolates harbored AMR genes, with the following distribution: 2% *bla*_VIM_, 5% *bla*_NDM-1_, 27% *bla*_NDM-5_, 65% *bla*_OXA_-type, and 1% *bla*_DIM-1_. Plasmid-associated AMR genes were detected in 58% of isolates; among these, 96% carried Inc-family plasmids, 57% Col plasmids, and 11% replication-associated elements (rep). Phylogenetic analysis demonstrated that certain isolates exhibited both hospital-specific and shared genetic profiles, indicating widespread dissemination across multiple healthcare facilities, as well as evidence of local emergence and ongoing transmission. **Conclusions:** The high prevalence of CR-GNB harboring resistance genes and plasmids underscores the urgent need for NGS-based genomic surveillance in South Lebanon. Implementing such strategies is essential for tracking resistance genes, identifying clonal outbreaks, and guiding effective infection control interventions to mitigate the spread of CR-GNB.

## 1. Introduction

Carbapenem-resistant Gram-negative bacteria (CR-GNB) represent an urgent public health threat, posing a significant risk to healthcare systems worldwide [1]. These organisms are capable of causing life-threatening infections associated with severe morbidity and mortality [2,3,4]. Outbreaks of CR-GNB within hospitals necessitate the implementation of critical infection control interventions, including patient isolation and the reinforcement of barrier precautions [5]. Patients in intensive care units (ICUs) are particularly vulnerable, as they frequently require invasive procedures, prolonged hospitalization, and extended courses of antibiotic therapy, all of which increase susceptibility to severe CR-GNB infections [6].

In Lebanon, insufficient antimicrobial resistance (AMR) surveillance and reporting have contributed to a marked increase in CR-GNB infections [7,8]. In fact, a 9 years surveillance study conducted at Saint Georges Hospital documents a significant rise in carbapenem-resistant *Acinetobacter baumannii*, *Pseudomonas aeruginosa*, and *Klebsiella pneumoniae* [7]. Moreover, broader analyses underscore that AMR surveillance in Lebanon remains limited, fragmented, and heavily reliant on single-center data, undermining timely detection and response to emergent CR-GNB trends [9]. Globally, next-generation sequencing (NGS) is increasingly utilized for pathogen identification, AMR gene detection, and epidemiological surveillance, owing to its high accuracy and rapid turnaround time [10].

Several molecular studies conducted across Lebanon have monitored carbapenem resistance in Gram-negative bacteria, revealing a diverse repertoire of resistance genes and mobile genetic elements [11,12]. Reported mechanisms include enzymatic degradation of antibiotics, the presence of efflux pump-associated genes, regulatory elements modulating resistance, and alterations in antibiotic targets [13].

This study aims to address gaps in the molecular surveillance of CR-GNB in Lebanon by investigating their prevalence and characterizing the distribution of resistance genes and plasmid types among ICU patients in hospitals in South Lebanon. Additionally, the study demonstrates the value of integrating NGS data with machine learning models to enhance the detection of carbapenemase gene patterns associated with high-risk CR-GNB outbreaks, thereby supporting and strengthening infection control measures.

## 2. Results

### 2.1. Overview

For reporting purposes, isolates classified as intermediate were considered resistant to provide a conservative estimate of resistance rates.

Out of 477 clinical isolates received from hospital laboratories, 131 were purified and identified as carbapenem-resistant Gram-negative bacteria (CR-GNB), corresponding to a prevalence of 27.4%. Isolates were obtained from various clinical specimens: swabs (67%), urine (13%), sputum (10%), deep tracheal aspirates (DTA; 9%), and blood (1%). Among these, *Escherichia coli* and *Pseudomonas aeruginosa* each accounted for 26.7% (*n* = 35), followed by *Klebsiella pneumoniae* (19.8%; *n* = 26), *Acinetobacter baumannii* (17.6%; *n* = 23), *Proteus mirabilis* (4.6%; *n* = 6), *Enterobacter cloacae* (2.3%; *n* = 3), *Achromobacter* spp. (1.5%; *n* = 2), and *Citrobacter freundii* (0.8%; *n* = 1). In Supplementary Table (Table S1), we summarize metadata for all sequenced isolates (*n* = 131): sample ID, BioProject Number, genus and species, sequence type, hospital of origin, specimen type, collection date, NCBI SRA accession numbers for deposited raw reads, in addition to raw meropenem disk diffusion data, i.e., inhibition zone diameters in mm.

### 2.2. Antimicrobial Resistance Rates of CR-GNB Isolates

**Figure 1:**
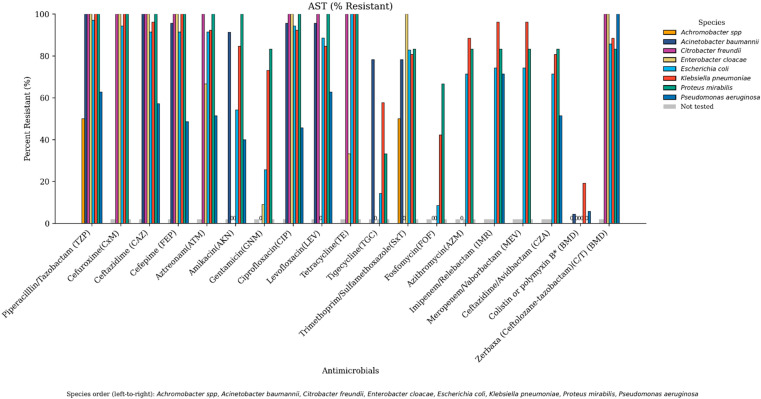
Antimicrobial resistance profiles of Gram-negative bacterial isolates across tested antimicrobials. Each bacterial species is represented by a distinct color in the following order: *Achromobacter* spp., *Acinetobacter baumannii*, *Citrobacter freundii*, *Enterobacter cloacae*, *Escherichia coli*, *Klebsiella pneumoniae*, *Proteus mirabilis*, and *Pseudomonas aeruginosa*. Antimicrobials not tested for a given species are shown in gray, reflecting agents not recommended by CLSI for that organism. Bars labeled with “0” indicate that all isolates of the corresponding species were sensitive to the tested antimicrobial.

A comprehensive overview of the antimicrobial susceptibility profile of CR-GNB across tested antimicrobial agents is depicted in Figure 1. The figure is organized by antibiotics to emphasize which agents exhibited the highest resistance rates across species, while the following narrative presents these results species by species to facilitate direct comparison among the major pathogens. All *A. baumannii* isolates (100%) exhibited resistance to meropenem, piperacillin–tazobactam and ceftazidime, while 95.7% were resistant to cefepime, levofloxacin, and ciprofloxacin. Moreover, 91.3% were resistant to amikacin. Furthermore, 78.3% were resistant to trimethoprim–sulfamethoxazole and tigecycline and 4.3% were resistant to colistin.

Resistance rates of *P. aeruginosa* isolates were as follows: 100% were resistant to meropenem and ceftolozane/tazobactam, 71.4% to imipenem/relebactam, 62.9% to piperacillin/tazobactam and levofloxacin, 57.1% to ceftazidime, 51.4% to aztreonam and ceftazidime–avibactam, 48.6% to cefepime, 45.7% to ciprofloxacin, 40% to amikacin, and 5.7% to colistin.

As for *E. coli*, 100% were resistant to both meropenem and tetracycline, 97.1% to piperacillin/tazobactam, 94.3% to ciprofloxacin and cefuroxime, 91.4% to ceftazidime, cefepime, and aztreonam, 88.6% to levofloxacin, 85.7% to ceftolozane–tazobactam, 82.9% to trimethoprim–sulfamethoxazole, 74.3% to imipenem–relebactam and meropenem–vaborbactam, 71.4% to ceftazidime–avibactam and azithromycin, 51.4% to amikacin, 25.6% to gentamicin, 14.3% to tigecycline, and 8.6% to fosfomycin. No resistance to colistin was observed.

*K. pneumoniae* isolates exhibited 100% resistance to meropenem, cefuroxime, ceftazidime, and piperacillin/tazobactam, 96.2% to cefepime, meropenem–vaborbactam and imipenem–relebactam, 92.3% to aztreonam and ciprofloxacin, 88.5% to ceftolozane–tazobactam and azithromycin, 84.6% to levofloxacin and amikacin, 80.8% to trimethoprim–sulfamethoxazole and ceftazidime–avibactam, 73.1% to gentamicin, 57.7% to tigecycline, 42.3% to Fosfomycin, and 19% to colistin.

**Figure 2:**
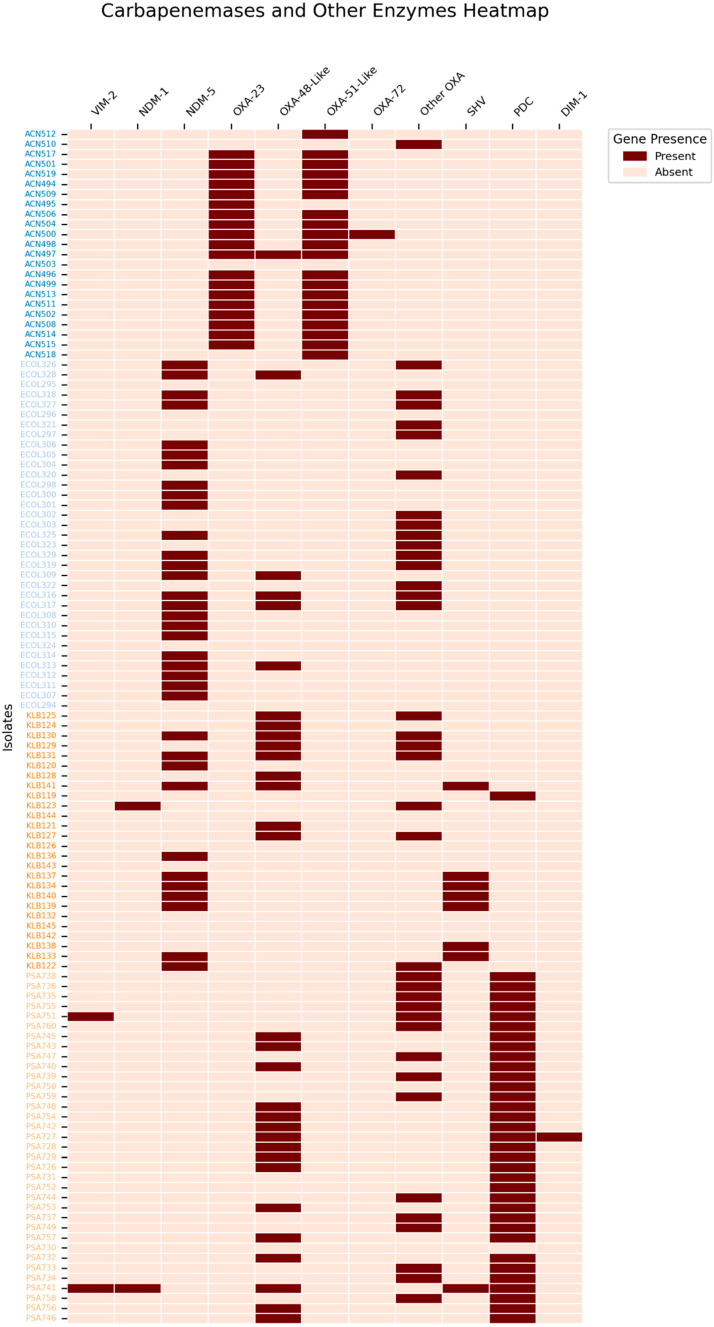
The distribution of carbapenemases and other key enzymes in the critical CR-GNB isolates, these include *Escherichia coli*, *Pseudomonas aeruginosa*, *Acinetobacter baumannii*, and *Klebsiella pneumoniae*. Other OXA variants include oxacillinases that do not contribute directly to carbapenem resistance, such as OXA-1, OXA-9, OXA-50, OXA-395, OXA-821, OXA-846, OXA-904, and OXA-908.

Antimicrobial susceptibility testing revealed that 0.8% (*n* = 1/131) of the isolates can be classified as pan-drug-resistant (PDR), 40.5% (*n* = 53/131) as extensively drug-resistant (XDR), and 52.7% (*n* = 69/131) as multi-drug-resistant (MDR). A heatmap was constructed to summarize the distribution of key AMR genes in carbapenem-resistant critical isolates such as *Escherichia coli*, *Pseudomonas aeruginosa*, *Acinetobacter baumannii*, and *Klebsiella pneumoniae* (Figure 2).

### 2.3. Carbapenem-Resistant Acinetobacter baumannii (CRAB)

CRAB comprised 17.6% (*n* = 23) of the total isolates. Phenotypic resistance profiling revealed that 4.3% (*n* = 1/23) were classified as PDR, 78.3% (*n* = 18/23) as XDR, and 17.4% (*n* = 4/23) as MDR. Multi-locus sequence typing (MLST) identified five sequence types, with ST2 predominating (65%), followed by ST717 (17%), ST154 (9%), ST19 (4%), and ST321 (4%). OXA-type carbapenemase genes were detected in 96% (*n* = 22/23) of CRAB isolates. Plasmid analysis identified rep-type plasmids in two isolates.

### 2.4. Carbapenem-Resistant Pseudomonas aeruginosa (CRPA)

CRPA accounted for 26.7% (*n* = 35) of CR-GNB isolates. Among these, 31.4% (*n* = 11/35) were classified as XDR, 48.6% (*n* = 17/35) as MDR, and 20% (*n* = 7/35) as AMR. The most frequently identified sequence type was ST274 (14%), followed by ST205, ST1498, and ST2105 (11% each); ST233, ST277, and ST1480 (9% each); ST412, ST111, and ST1428 (6% each); ST175 and ST1047 (3% each); and one isolate of unknown ST. Two isolates harbored the *bla*_VIM_ gene and one isolate harbored *bla*_NDM-1_. OXA-type carbapenemase-encoding genes were present in 97% (*n* = 34/35) of CRPA isolates. Inc-type and Col-type plasmids were detected in 75% of isolates.

### 2.5. Carbapenem-Resistant E. coli (CREC)

CREC comprised 26.7% (*n* = 35) of CR-GNB isolates. Phenotypic analysis indicated that 14.3% (*n* = 5/35) were XDR and 85.7% (*n* = 30/35) MDR. MLST revealed a diverse distribution: ST167 (29%), ST2 (9%), ST38 (6%), ST46 (6%), and several other STs each accounting for 3%. Unknown STs constituted 9%. The *bla*_NDM-5_ gene was present in 69% (*n* = 24/35) of isolates, and OXA-type carbapenemases in 46% (*n* = 16/35). All CREC isolates harbored Inc-type plasmids (100%), with Col-type in 40% and rep-type in 3%.

### 2.6. Carbapenem-Resistant Klebsiella pneumoniae (CRKP)

CRKP accounted for 19.8% (*n* = 26) of CR-GNB isolates. Resistance profiles showed that 53.8% (*n* = 14/26) were classified as XDR and 46.2% (*n* = 12/26) as MDR. The most prevalent ST was ST147 (35%), followed by ST39 (19%), ST25 (15%), ST14 (8%), ST15, ST28, ST252, and ST5118 (each 4%), and 8% with unknown ST. The most prevalent carbapenemase genes were *bla*_OXA-48_-like (46%; *n* = 12/26), followed by *bla*_NDM-5_ (42%; *n* = 11/26) and *bla*_NDM-1_ (4%; *n* = 1/26). Plasmid analysis showed Inc-type plasmids in all isolates (100%), Col-type in 69%, and rep-type in 8%.

### 2.7. Carbapenem-Resistant Proteus mirabilis

Carbapenem-resistant *P. mirabilis* represented 4.6% (*n* = 6) of CR-GNB isolates. Of these, 66.7% (*n* = 4/6) were classified as XDR and 33.3% (*n* = 2/6) as MDR. All isolates were of unknown ST. *bla*_NDM-5_ and OXA-type carbapenemase-encoding genes were each detected in 16.7% (*n* = 1). Plasmid analysis revealed Inc- and Col-type plasmids in 83% of isolates and rep-type in 17%.

### 2.8. Other CR-GNB

#### 2.8.1. *Enterobacter cloacae*

All three isolates were classified as MDR and belonged to ST1377. These were recovered from DTA (*n* = 1) and swabs (*n* = 2). Moreover, all were positive for the *bla*_NDM-1_ gene and harbored both Inc- and Col-type plasmids.

#### 2.8.2. *Achromobacter* spp.

Two isolates were identified, one of which was MDR. One belonged to ST283 while the other was of unknown ST, recovered from breast swab and sputum.

#### 2.8.3. *Citrobacter freundii*

A single MDR isolate belonging to ST112 was recovered from a nasal swab and was positive for the *bla*_NDM-1_ gene. An Inc-type plasmid was detected.

### 2.9. Phylogenetic Tree

**Figure 3:**
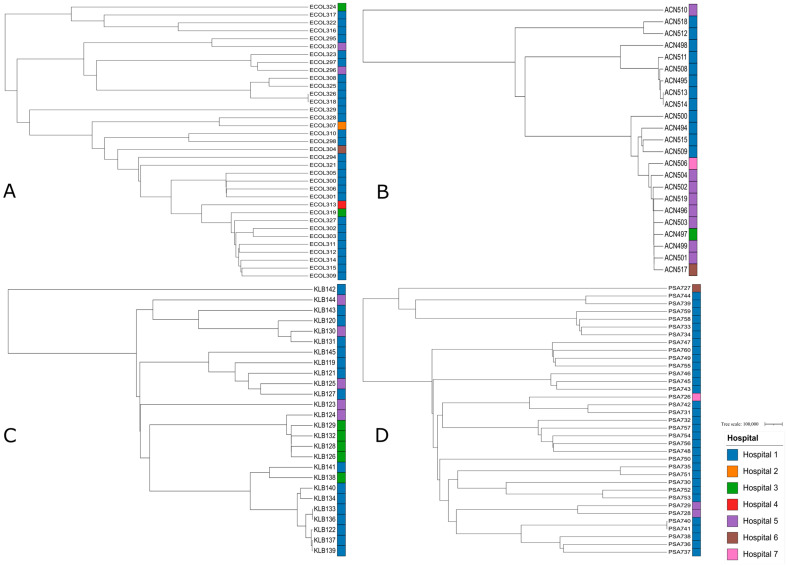
A total of 4 phylogenetic trees with branch length of the critical isolates, color-labeled by the hospitals they were isolated from, showing the genetic relatedness between those. Tree A represents the phylogenetic tree of *Escherichia coli* isolates. Tree B represents the phylogenetic tree of *Pseudomonas aeruginosa* isolates. Tree C represents the phylogenetic tree of *Acinetobacter baumannii* isolates. Tree D represents the phylogenetic tree of *Klebsiella pneumoniae* isolates.

Phylogenetic trees were generated for the four main Gram-negative species recovered in this study: *E. coli*, *P. aeruginosa*, *A. baumannii*, and *K. pneumoniae* (Figure 3). This approach efficiently elucidated the evolutionary relationships among the carbapenem-resistant Gram-negative bacterial isolates studied. In fact, isolates from the same species and the same hospital often clustered together ex: *Acinetobacter baumannii* isolates ACN511, ACN508, ACN495, ACN513, and ACN514 coming from hospital 1, *Escherichia coli* isolates ECOL309, ECOL315 with ECOL314, and ECOL312 coming from hospital 1, *Pseudomonas aeruginosa* isolates PSA748, PSA756, and PSA754 from hospital 1, and *Klebsiella pneumoniae* isolates KLB126, KLB128, KLB132, and KLB129 coming from hospital 3. Moreover, several isolates were found to cluster within clades otherwise confined to a single hospital such as ECOL307 from hospital 2 clustering with a clade dominated by isolates from hospital 1, PSA726 showing genetic similarities to PSA742 and PSA731 isolated from hospital 1, ACN497 clustering with *Acinetobacter baumannii* isolates coming from hospital 5, and KLB124 from hospital 5 clustering with a clade of isolates coming from hospital 3.

## 3. Discussion

Given that most molecular epidemiology reports of carbapenem-resistant Gram-negative bacteria in the country have originated from Beirut and the northern governorates, hospital-based genomic data from southern Lebanon remain scarce. In particular, outbreak and surveillance studies from tertiary centers in Beirut and multi-hospital investigations in the North are predominant, leaving a critical geographic gap for the South that this study begins to address [12,14,15].

In our study, among all CR-GNB isolates collected, 17.6% were *A. baumannii*, 19.8% were *K. pneumoniae*, 26.7% were *P. aeruginosa*, and 26.7% were *E. coli*. This distribution reveals a more balanced spread of carbapenem resistance across species compared to several previous reports, where *A. baumannii* or *K. pneumoniae* typically dominate the CR-GNB profile. For instance, a multi-country review by Davoudi-Monfared and Khalili [16] highlighted *A. baumannii* as the leading CR-GNB species across the Middle East, often comprising 60–85% of resistant isolates, particularly in ICU and burn units. Similarly, other global studies have reported *K. pneumoniae* as a primary contributor to carbapenem resistance in hospital settings [16].

CRAB represented 17.6% of CR-GNB included in our study, with resistance largely mediated by OXA-type carbapenemases and a predominance of international clone II (IC2/ST2). These features mirror Lebanese hospital data showing IC2/ST2 dominance and OXA-23 carriage during ICU outbreaks, and are concordant with more recent reports documenting XDR/PDR phenotypes in Lebanese ICUs [15]. Across the Eastern Mediterranean Region (EMR), systematic syntheses similarly identify OXA-23 as the leading CRAB carbapenemase and IC2 as the dominant lineage underpinning hospital transmission [17]. Globally, IC2 is the most widespread CRAB lineage and OXA-23 remains a principal driver of carbapenem resistance; CRAB is classified among the highest-threat organisms in the 2024 WHO Bacterial Priority Pathogens List, underscoring the clinical gravity of these findings [3,18].

CRPA accounted for 26.7% of CR-GNB and showed diverse STs with detection of VIM metallo-β-lactamases. Lebanese and EMR reports document VIM as a recurrent CRPA carbapenemase, alongside non-enzymatic mechanisms, porin loss (notably OprD), and overactive RND efflux systems, synergize with acquired enzymes to raise carbapenem MICs [14]. Outside the EMR, VIM-producing CRPA has been central to notable outbreaks. In the United States, a 2022–2023 multistate cluster linked to contaminated artificial tears involved VIM-GES CRPA with substantial morbidity; European surveillance likewise tracks sustained CRPA burdens and variable carbapenem resistance across member states [19].

As for our carbapenem-resistant *Escherichia coli* (CREC) isolates, they accounted for 26.7% of total isolates, indicating a higher local burden compared with data from northern Lebanon [14]. ST167 was the dominant sequence type (29%), corroborating its status as a high-risk clone associated with global dissemination of carbapenem resistance, particularly across Asia and North America [20]. The elevated prevalence of *bla*_NDM-5_ (69%) and OXA-type carbapenemases (46%) among CREC aligns with recent Lebanese studies documenting the spread of *bla*_NDM_-positive *E. coli* [21]. Of particular concern, all CREC isolates harbored Inc-type plasmids, which are frequently implicated in the horizontal transfer of *bla*_NDM-5_ and OXA-type carbapenemases, thereby facilitating rapid dissemination of resistance. At the regional level, data from the EMR indicate increasing NDM prevalence among *Enterobacterales*, including *E. coli*, with plasmid-mediated dissemination [4]. Globally, NDM-5 has rapidly expanded across Europe, with cross-border transmission documented through 2012–2022 surveillance; ST167 is repeatedly implicated as a vehicle for NDM-5 dissemination [22,23].

A systematic review of 61 articles across 14 countries found hospital-acquired carbapenem-resistant *Klebsiella pneumoniae* (CRKP) infections in ICUs reaching as high as 62.3%; in contrast, our observed prevalence (20%) is markedly lower [24]. Nevertheless, the predominance of the high-risk clone ST147 (35%) is alarming, given its established association with carbapenem resistance dissemination. High frequencies of *bla*_NDM-5_- (42%) and *bla*_OXA-48_-like (46%) genes in CRKP further highlight the role of plasmid-mediated horizontal gene transfer in the transmission of these resistance determinants within ICU settings. Moreover, Lebanese and EMR data consistently identify OXA-48-like and NDM as principal CRKP drivers, in line with our molecular findings [12]. Beyond the EMR, Europe continues to report a deteriorating epidemiologic situation for CRE/CRKP with expansion of high-risk lineages (e.g., ST258/512, ST11, ST101, ST307, and ST147) and dynamic mixtures of KPC, OXA-48-like, and NDM across countries; surveillance indicates rising carbapenem-resistant *K. pneumoniae* bloodstream infections at the EU/EEA level. In the United States, historically KPC-predominant, public health alerts now note a sharp increase in NDM-positive CRE, highlighting the growing global footprint of NDM [24,25,26].

A study on the emergence of Gram-negative bacteria in a hospital in Jordan reported that carbapenem-resistant *Proteus mirabilis* accounted for only 0.2% (24 isolates) of all Gram-negative infections [27]. In contrast, our findings revealed a higher prevalence of 4.6% among CR-GNB isolates in South Lebanon, an alarmingly higher occurrence compared to a neighboring country. While *bla*_NDM-5_ was detected, these genes remain uncommon in this species. The widespread presence of plasmids across isolates underscores a significant potential for intra- and interspecies horizontal gene transfer, consistent with global concerns [28].

Three carbapenem-resistant *Enterobacter cloacae* isolates, all belonging to ST1377, were identified, suggesting potential nosocomial transmission of a clonal strain. This finding parallels reports of nosocomial clusters involving plasmid-encoded *bla*_NDM-1_-producing *E. cloacae* in healthcare settings [29]. In addition, two carbapenem-resistant *Achromobacter* spp. isolates were detected, one belonging to ST283 and the other untyped, both lacking plasmids. This observation aligns with international studies indicating that carbapenem resistance in *Achromobacter* is often attributed to chromosomal genes and regulatory mechanisms rather than plasmid-mediated acquisition via chromosomal OXA-114-like class D β-lactamases and RND-type efflux, rather than driven by acquired carbapenemases [30,31]. Additionally, a single *Citrobacter freundii* isolate (ST112) was detected, a sequence type recognized as an emerging multidrug-resistant pathogen in the region and of significant clinical concern [32].

Despite the inclusion of distinct patient populations across different hospitals, the isolates demonstrated both hospital-specific and shared genetic patterns, suggesting that regional factors may be influencing bacterial evolution [33]. Regional pressures may drive convergent genetic adaptations, as evidenced by phylogenetic clustering of closely related isolates within specific hospitals. These patterns likely reflect selective pressures unique to each institution, such as differing antimicrobial stewardship policies and infection control measures, resulting in local strain adaptation [34].

Clustering of isolates from multiple hospitals within the same phylogenetic branches further indicates regional circulation of diverse strains, potentially mediated by both healthcare- and community-associated transmission [35].

## 4. Conclusions

In this first genomic survey of carbapenem-resistant Gram-negative bacteria (CR-GNB) from ICUs in southern Lebanon, our 131 carbapenem-resistant clinical isolates had a balanced species distribution: *Pseudomonas aeruginosa* and *Escherichia coli* (each 26.7%), *Klebsiella pneumoniae* (19.8%), and *Acinetobacter baumannii* (17.6%). Whole-genome sequencing analysis showed predominant species-specific associations of sequence types with carbapenem-resistance determinants, namely OXA-type genes with ST2 in CRAB; VIM in a subset of CRPA within a genetically diverse background; NDM-5 frequently in CREC with representation of the ST167 high-risk lineage; and concurrent OXA-48-like and NDM in CRKP with prevalence of ST147. Plasmid replicons were common, notably Inc and Col families, supporting a role for horizontal gene transfer alongside clonal spread in the dissemination of carbapenem resistance among CR-GNB. Phylogenetic clustering revealed both hospital-specific and cross-hospital shared genetic patterns, consistent with intra- and inter-facility transmission. Phenotypically, most isolates were MDR or XDR, with a small PDR fraction, underscoring constrained therapeutic options. Together, these findings indicate that carbapenem resistance in the region is sustained by a species-specific combination of high-risk sequence types, prominent carbapenemases and mobile genetic elements, necessitating routine, WGS-based surveillance integrated with infection-prevention programs and antimicrobial stewardship. Such coordinated, genomic-informed control efforts are likely to be crucial for detecting transmission, interrupting plasmid-mediated spread, and guiding empiric therapy in southern Lebanon and comparable settings.

## 5. Materials and Methods

### 5.1. Sample Collection

A total of 477 CR-GNB were collected from 10 Lebanese hospitals in the South, between January 2023 and December 2023. Due to Lebanon’s healthcare challenges, some hospitals conduct surveillance programs, while others rely on clinical cultures based on patient conditions, especially in high-risk ICU settings or for patients with known CR-GNB colonization. To ensure a comprehensive analysis, we included isolates from both surveillance and clinically indicated cultures in our study, allowing us to assess the distribution of CR-GNB and transmission dynamics, while also characterizing their genetic profiles.

All clinical isolates were recovered as part of the routine bacteriology workflow at selected hospitals in South Lebanon. Gram-negative bacterial specimens (*n* = 477) from different laboratories were collected and processed at the American University of Beirut–Bacteriology and Molecular Microbiology Research Laboratory. Samples were cultured and purified on MacConkey agar (Neogen, Lansing, MI, USA). Isolates were then selected on MacConkey supplemented with 2 mg/L meropenem and CHROMagar™ mSuperCARBA™ (CHROMagar, La Plaine St-Denis, France). Isolates that showed growth on both media (*n* = 131) were stored at −80 °C in a 25% glycerol solution for further analysis. These isolates originated from 7 out of the 10 included hospitals

### 5.2. Antimicrobial Susceptibility Testing (AST)

Antimicrobial susceptibility of different bacterial species was determined using the Kirby–Bauer disk diffusion (DD) method on Mueller–Hinton agar against a panel of antimicrobials except colistin and ceftolozane–tazobactam (zerbaxa), which were tested using broth microdilution (BMD). Selection of Antimicrobials was performed according to the Clinical and Laboratory Standards Institute M100 Edition 34 (CLSIM100 Ed34) [36]. Moreover, Tigecycline was also considered for *Enterobacterales* and *A. baumannii* due to its high clinical relevance in treating MDR infections [37]. Species-specific selection of antimicrobials is shown in Table 1. *Escherichia coli* ATCC^®^ 25922 and *Acinetobacter baumannii* DSM 30008 strains were both used as controls. The diameters of the inhibition zones and the minimum inhibitory concentrations (MICs) were determined and interpreted according to the Clinical and Laboratory Standards Institute CLSIM100 Ed34 for all antimicrobial except for Tigecycline which was interpreted according to EUCAST 2024 guidelines [36,38].

### 5.3. DNA Extraction

Bacterial genomic DNA was extracted from bacterial strains cultured on MacConkey agar using the Quick-DNA™ Fungal/Bacterial Miniprep kit (Zymo Research, Irvine, CA, USA), then DNA was purified using the Genomic DNA Clean and Concentrator™ kit (Zymo Research, Irvine, CA, USA) according to the manufacturer’s protocols.

### 5.4. Library Preparation and Sequencing

Short-Read Whole-Genome Sequencing: DNA library preparation was performed using the Illumina DNA prep kit (Illumina, San Diego, CA, USA), according to Illumina’s reference guide. Briefly, DNA underwent an initial tagmentation step followed by post-tagmentation cleanup. Then, tagmented DNA was amplified with addition of DNA/RNA unique dual (UD) indexes (Illumina) followed by library cleanup and elution. The concentrations of DNA libraries were measured using the Qubit dsDNA High Sensitivity assay kit (Invitrogen, Waltham, MA, USA) on a Qubit 4 fluorometer (Invitrogen). DNA libraries were pooled, denatured, and diluted to 12 pM. PhiX Control v3 (Illumina) was added, then the pooled library was sequenced using a MiSeq V2 Reagent Kit (500 cycles) on an Illumina MiSeq platform (Illumina) for 250 × 2 cycles and achieved a 50× coverage.

Long-Read Whole-Genome Sequencing: DNA library preparation was performed using the rapid barcoding kit (V14) (Oxford Nanopore Technologies (ONT), Oxford, UK) according to ONT’s protocol. Briefly, bacterial genomic DNA underwent barcoding, pooling then cleanup. Then, the resulting DNA libraries were eluted and their concentrations were measured on a Qubit 4 fluorometer (Invitrogen). Finally, the DNA libraries were loaded on an R10.4.1 flow cell (ONT) and sequenced on an Mk1B device (ONT).

### 5.5. Bioinformatic Analysis

Reads quality control and trimming were performed using Trimmomatic (v.1.2.14), after which assembly of the genome was performed using Unicycler on the UseGalaxy platform https://usegalaxy.org/ (accessed on 26 June 2024). Antimicrobial resistance genes were acquired through CARD https://card.mcmaster.ca/ (accessed on 28 June 2024). Sequence Types were identified using multi-locus sequence typing (MLST) on UseGalaxy. Plasmid replicons were identified and characterized using PlasmidFinder https://cge.food.dtu.dk/services/PlasmidFinder/ (accessed on 27 June 2024). KmerFinder was used for rapid species identification of the bacterial isolates. Phylogenetic tree was generated using SaffronTree, a kmer-based method to classify bacterial isolates by comparing k-mer frequency distributions. Phylogenetic trees were visualized using iTOL v6 (Interactive Tree of Life), an online tool for displaying, color-coding, and customizing phylogenetic trees. Metadata including hospital origin was added using color strip datasets, and trees were exported in vector format for figure integration.

### 5.6. Data Availability

The accession numbers of the samples included in the analysis are presented in Supplementary Table (Table S1).

## Figures and Tables

**Table 1:** Antimicrobial agents tested against each bacterial species included in the study. List of antimicrobial agents used for susceptibility testing of each bacterial species. Testing was performed using the Kirby–Bauer disk diffusion method for most agents, except colistin and ceftolozane–tazobactam, which were tested by broth microdilution. Antimicrobial selection was based on CLSI M100 (34th ed.), with tigecycline included due to its clinical significance.

Method	Class	Agent	*Escherichia coli*	*Klebsiella pneumoniae*	*Pseudomonas aeruginosa*	*Acinetobacter baumannii*	*Enterobacter cloacae*	*Citrobacter freundii*	*Proteus mirabilis*	*Achromobacter* spp.
Disk Diffusion	Carbapenems	Meropenem (10 µg)	✓	✓	✓	✓	✓	✓	✓	
	Cephalosporins	Cefuroxime (30 µg)	✓	✓			✓	✓	✓	
		Ceftazidime (30 µg)	✓	✓	✓	✓	✓	✓	✓	
		Cefepime (30 µg)	✓	✓	✓	✓	✓	✓	✓	
	Monobactam	Aztreonam (30 µg)	✓	✓	✓		✓	✓	✓	
	β-Lactams/β-Lactamase Combination	Ceftazidime–avibactam (30/20 µg)	✓	✓	✓		✓	✓	✓	
		Imipenem–relebactam (10/25 µg)	✓	✓	✓		✓	✓	✓	
		Piperacillin–tazobactam (100/10 µg)	✓	✓	✓		✓	✓	✓	✓
		Meropenem–vaborbactam (20/10 µg)	✓	✓			✓	✓	✓	
	Fluoroquinolones	Ciprofloxacin (5 µg)	✓	✓	✓	✓	✓	✓	✓	
		Levofloxacin (5 µg)	✓	✓	✓	✓	✓	✓	✓	
	Aminoglycosides	Amikacin (30 µg)	✓	✓	✓	✓	✓	✓	✓	
		Gentamicin (10 µg)	✓	✓		✓	✓	✓	✓	
	Tetracyclines	Tetracycline (30 µg)	✓	✓			✓	✓	✓	
	Glycylclines	Tigecycline (15 µg) *	✓	✓		✓	✓	✓	✓	
	Phosphonic acid Derivatives	Fosfomycin (200 µg)	✓	✓			✓	✓	✓	
	Macrolides	Azithromycin (15 µg)	✓	✓			✓	✓	✓	
	Combination of Diaminopyrimidines and Sulfonamides	Trimethoprim–Sulfamethoxazole (1.25/23.75 µg)	✓	✓		✓	✓	✓	✓	✓
Broth Microdilution	Combination of Cephalosporins and β-lactamase inhibitors	Ceftolozane–tazobactam (1:2)	✓	✓	✓		✓	✓	✓	
	Polymyxins	Colistin	✓	✓	✓	✓	✓	✓	✓	✓

✓ = antibiotic tested; Blank = antibiotic not tested; * = Antimicrobial agents tested against each bacterial species included in the study. List of antimicrobial agents used for susceptibility testing of each bacterial species. Testing was performed using the Kirby–Bauer disk diffusion method for most agents, except colistin and ceftolozane–tazobactam, which were tested by broth microdilution. Antimicrobial selection was based on CLSI M100 (34th ed.), with tigecycline included due to its clinical significance.

## Data Availability

Any inquiries can be directed to the corresponding author.

## Supplementary Materials

The following supporting information can be downloaded at: https://www.mdpi.com/article/10.3390/antibiotics14111124/s1; Table S1: Metadata for sequenced CR-GNB isolates.

## Sequence Information

The WGS data of the isolates analyzed in this work have been deposited in the National Center for Biotechnology Information (NCBCI) database under BioProject number PRJNA613441. Accession numbers are listed in Supplementary Table (Table S1).

## Abbreviations

The following abbreviations are used in this manuscript:

AMR	Antimicrobial Resistance
CARD	Comprehensive Antibiotic Resistance Database
CR-GNB	Carbapenem-resistant Gram-negative bacteria
ICU	Intensive Care Unit
MLST	Multi Locus Sequence Typing
NGS	Next-Generation Sequencing
CLSI	Clinical and Laboratory Standards Institute
EUCAST	European Committee on Antimicrobial Susceptibility Testing.
